# Haemoglobin concentration following postpartum haemorrhage and the association between blood transfusion and breastfeeding: a retrospective cohort study

**DOI:** 10.1186/s13104-018-3800-0

**Published:** 2018-10-01

**Authors:** Julia Chessman, Jillian Patterson, Tanya Nippita, Bradley Drayton, Jane Ford

**Affiliations:** 1 Clinical and Population Perinatal Health Research, Kolling Institute, Northern Sydney Local Health District, St Leonards, NSW Australia; 2 The University of Sydney Northern Clinical School, St Leonards, NSW Australia; 3 NSW Biostatistics Training Program, NSW Ministry of Health, North Sydney, Australia; 4 Department of Obstetrics and Gynaecology, Royal North Shore Hospital, St Leonards, NSW Australia

**Keywords:** Postpartum haemorrhage, Haemoglobin concentration, Red blood cell transfusion, Breastfeeding

## Abstract

**Objective:**

The aim of this study was to determine the association between red blood cell transfusion and breastfeeding among women who have suffered a postpartum haemorrhage at birth taking into account post-birth haemoglobin concentrations.

**Results:**

Among 15,451 maternities with postpartum haemorrhage in New South Wales public hospitals between 2007 and 2010, 1828 (12%) received a red cell transfusion. Among transfused women, 686 (38%) had haemoglobin concentration pre-transfusion < 70 g/L, 792 (43%) had 70–90 g/L, and 350 (19%) had > 90 g/L. Rates and adjusted relative risks (aRR) for breastfeeding at hospital discharge were as follows: for women with haemoglobin concentrations < 70 g/L following birth and received a transfusion, 78.6% were breastfeeding and the aRR of breastfeeding compared to untransfused women was 0.90 (99% confidence interval (CI) 0.86–0.95); for women with haemoglobin concentrations 70–90 g/L, 81.3% were breastfeeding, aRR 0.94 (99% CI 0.90–0.98); and for women with haemoglobin concentrations > 90 g/L, 80.9% were breastfeeding, aRR 0.94 (99% CI 0.88–1.00).

## Introduction

Postpartum haemorrhage rates and transfusion rates following postpartum haemorrhage have been increasing in high resource settings [1–3]. In New South Wales (NSW), Australia the postpartum haemorrhage rate has recently been estimated at 8.3% and postpartum haemorrhage with transfusion at 1.2% of births [3].

In the postpartum anaemic woman without ongoing bleeding, the decision to transfuse may not be straightforward. Clinicians must weigh the benefits to energy levels against the risks of transfusion such as bloodborne infections, transfusion reactions and immunologic effects [4]. Pregnant and recently pregnant women are generally healthy and can tolerate some anaemia, however there are other factors to consider including caring for a new baby.

Breastfeeding is an important aspect of early infant care, with benefits for both infant and maternal health [5]. Studies have shown that postpartum anaemia is associated with insufficient milk supply and early breastfeeding cessation [6, 7]. Anecdotally, some clinicians believe that transfusion may increase the likelihood of breastfeeding in postpartum anaemic woman [8].

An analysis of breastfeeding rates at discharge in women with postpartum haemorrhage in NSW hospitals found that transfusion was associated with a lower rate of breastfeeding [9]. After adjustment for clinical and demographic factors, Drayton et al. found the rate of any breastmilk feeding at discharge to be 0.94 (99% confidence interval (CI) 0.92, 0.95) in transfused vs non-transfused women. At the time, data were not available to indicate the severity of the haemorrhage and the extent to which this influenced the findings. Data on haemoglobin concentration pre- and post-transfusion have since become available for a subgroup of the NSW birthing population.

The aim of this study is to explore the association between red blood cell transfusion and breastfeeding among women who have suffered a postpartum haemorrhage taking into account haemoglobin concentrations pre-transfusion.

## Main text

### Methods

The study population included maternities where the mother suffered a postpartum haemorrhage (defined as blood loss of at least 500 mL post-vaginal birth and at least 750 mL post-caesarean birth) following a singleton birth of at least 37 weeks gestation where the mother and baby were alive at hospital discharge. The cohort was further restricted to births that occurred in a NSW public hospital that submitted blood transfusion data to the NSW Clinical Excellence Commission (CEC) Blood Watch program between January 2007 and December 2010.

Data were drawn from the NSW Perinatal Data Collection (PDC), the NSW Admitted Patient Data Collection (APDC) and the CEC Blood Watch program database. These datasets were probabilistically linked by the Centre for Health Record Linkage (CHeReL) [10]. The PDC is a statutory data collection of all births in NSW of at least 20 weeks gestation or 400 g birth weight and contains information on the pregnancy, labour and delivery. The APDC is a census of all patient admissions in NSW hospitals and contains up to 50 diagnoses and procedures coded from the medical record. The CEC Blood Watch program database contains information on red blood cell transfusion and pathology results in NSW public hospitals between July 2006 and December 2010. Hospital participation in the program was staggered over time. In the months when a hospital was participating in the program, information on each blood pack issued and the results of haemoglobin pathology tests were submitted to the CEC Blood Watch program. About half of all births in NSW between July 2006 and December 2010 occurred in a public hospital that was submitting data to the CEC Blood Watch program. This subset is representative of the NSW public hospital maternity population, but marginally higher risk compared to the NSW maternity population overall [11].

The study exposure was red blood cell transfusion following postpartum haemorrhage and the outcome was any breastfeeding at hospital discharge. Maternities were excluded from the analysis if the mother was admitted to intensive care or transfused with other blood products (platelets, coagulation factors or serum). These factors suggest that the mother suffered a severe haemorrhage and were excluded to make the transfused and non-transfused groups more comparable. Maternities where the mother was transfused during the birth admission but prior to the baby’s date of birth, or transfused but did not have a haemoglobin measure on or up to 2 days prior to transfusion, were also excluded.

Postpartum haemorrhage and transfusion of red blood cells or other blood products were identified using diagnosis and procedure codes in hospital records. Ascertainment of these conditions and procedures is high in hospital data [12]. If a mother did not have a transfusion procedure code in hospital data but was issued blood during their hospital admission (according to CEC Blood Watch data); they were also classified as transfused. The blood pack issue date and the haemoglobin order date in CEC Blood Watch data were taken as the date of transfusion and the date of haemoglobin measure respectively. Baby feeding status at discharge—breastfeeding, expressed breast milk and/or infant formula—was obtained from birth records. Mothers were considered to be exclusively breastfeeding if either or both of the first two options were selected, and partially breastfeeding if infant formula was selected in addition to either or both of the first two options. Exclusive and partial breastfeeding were combined into a single category, ‘any breastfeeding’, for analysis.

Poisson regression models with sandwich error estimation [13] were used to explore the relationship between transfusion and breastfeeding among maternities with postpartum haemorrhage. The analysis was stratified by haemoglobin concentration prior to transfusion—less than 70 g/L, 70–90 g/L and greater than 90 g/L as per haemoglobin ranges used in maternity guidelines [14]. We used the lowest haemoglobin measure on or up to 2 days prior to transfusion to stratify results. Each haemoglobin-transfusion group was compared to the entire non-transfused group. Descriptive statistics on haemoglobin measures after transfusion but before hospital discharge were produced for each haemoglobin-transfusion group. Haemoglobin measures were not available for the non-transfused group.

Factors included in modelling to control for confounding were: maternal age, country of birth, marital status, parity, hypertension, diabetes, smoking during pregnancy, public/private patient status, analgesia for labour and delivery, mode of delivery, gestational age, large for gestational age (> 90th percentile of birth weight, given gestational age), small for gestational age (< 10th percentile of birth weight, given gestational age) [15], resuscitation procedures, Apgar score at 5 min less than seven, transfer of baby, admission to special care nursery or neonatal intensive care unit, maternal morbidity [16] (a composite measure of morbidity but in this case excluding transfusions and procedures limiting blood flow to the uterus), maternal hospital classification [17], year of birth, and length of the birth admission from date of birth. These variables have high validity [12, 18]. Records with missing covariate information were excluded from the regression analysis. Purposeful selection [19] was followed with variables significant at the 0.01 level or variables that when excluded caused a greater than 10% change in coefficients retained in the model.

Ethics approval was obtained from the NSW Population and Health Services Research Ethics Committee. The analyses were performed in SAS 9.3.

### Results

There were 16,504 maternities with postpartum haemorrhage in our study population. Four percent (n = 636) of maternities were excluded based on an indication of a severe postpartum haemorrhage (intensive care admission or other blood products transfused). A further 19 maternities were excluded for a transfusion prior to the baby date of birth. Among the 2226 (14%) maternities involving red blood cell transfusion, 398 (18%) were excluded because they did not have a haemoglobin measure on or up to 2 days prior to transfusion. The final cohort of 15,451 maternities with postpartum haemorrhage included 1828 transfused mothers (1691 identified in APDC, additional 137 identified in CEC Blood Watch data) and 13,623 non-transfused mothers. Covariate information was missing for 126 maternities and they were excluded from regression analysis. Among transfused mothers, 686 (38%) had haemoglobin concentration pre-transfusion < 70 g/L, 792 (43%) had 70–90 g/L and 350 (19%) had > 90 g/L.

A higher percentage of the transfused mothers were primiparous compared to the non-transfused mothers (55% vs 49%) (Table 1). Among the transfused mothers, primiparous women were over-represented in the lowest haemoglobin group. Maternal morbidity [16] was about six times higher in transfused mothers compared to non-transfused mothers (6% vs 1%). Among the transfused mothers, the rate of maternal morbidity was slightly higher in the haemoglobin group > 90 g/L compared to the other two groups. The babies of transfused mothers were more likely to be admitted to a special care nursery or a neonatal intensive care unit compared to the babies of non-transfused mothers (20% vs 14%).

**Table 1:** Demographic and clinical characteristics of maternities with postpartum haemorrhage by transfusion and haemoglobin concentration prior to transfusion

	Transfusion						No transfusion	
	Haemoglobin < 70 g/L		Haemoglobin 70–90 g/L		Haemoglobin > 90 g/L			
	n	col%^a^	n	col%^a^	n	col%^a^	n	col%^a^
Maternities	686		792		350		13,623	
Maternal age								
19 and under	29	4.2	28	3.5	8	2.3	285	2.1
20–34	550	80.2	629	79.4	266	76.0	10,715	78.7
35 and over	107	15.6	135	17.0	76	21.7	2622	19.2
Country of birth								
Australia	394	57.4	526	66.4	223	63.7	7987	58.6
Asia	165	24.1	146	18.4	61	17.4	2718	20.0
Other and not stated	127	18.5	120	15.2	66	18.9	2918	21.4
Parity								
Primipara	424	61.8	427	53.9	159	45.4	6611	48.5
1–2 previous births	203	29.6	290	36.6	157	44.9	5696	41.8
3+ previous births	58	8.5	73	9.2	34	9.7	1303	9.6
Hypertension	94	13.7	92	11.6	54	15.4	1191	8.7
Diabetes	47	6.9	46	5.8	30	8.6	1093	8.0
Smoked during pregnancy	98	14.3	118	14.9	47	13.4	1624	11.9
Private patient	69	10.1	96	12.1	42	12.0	1472	10.8
Analgesia—spinal/epidural	320	46.6	352	44.4	139	39.7	5497	40.4
Analgesia—general anaesthetic	139	20.3	158	19.9	83	23.7	942	6.9
Analgesia—opioid	170	24.8	192	24.2	70	20.0	2967	21.8
Delivery mode								
Vaginal (including breech)	386	56.3	434	54.8	207	59.1	8694	63.8
Instrumental birth	189	27.6	183	23.1	62	17.7	2376	17.4
Caesarean—labour	76	11.1	121	15.3	49	14.0	1574	11.6
Caesarean—no labour	35	5.1	53	6.7	32	9.1	957	7.0
Maternal morbidity	39	5.7	45	5.7	26	7.4	117	0.9
Placenta praevia/accreta	29	4.2	41	5.2	32	9.1	222	1.6
Estimated gestational age (weeks)								
37–38	148	21.6	149	18.8	86	24.6	2567	18.8
39–40	364	53.1	439	55.4	177	50.6	7795	57.2
41+	174	25.4	204	25.8	87	24.9	3261	23.9
Large for gestational age	96	14.0	131	16.5	54	15.4	2012	14.8
Small for gestational age	42	6.1	47	5.9	25	7.1	963	7.1
Resuscitated	67	9.8	81	10.2	43	12.3	1102	8.1
Apgar score < 7 at 5 min	13	1.9	15	1.9	9	2.6	200	1.5
Transfer of baby	9	1.3	13	1.6	10	2.9	311	2.3
Admitted to SCN or NICU	125	18.2	153	19.3	80	22.9	1898	13.9
Breastfeeding at discharge								
No breastfeeding	147	21.4	148	18.7	67	19.1	1761	12.9
Exclusive	447	65.2	562	71.0	235	67.1	10,740	78.8
Partial	92	13.4	82	10.4	48	13.7	1122	8.2
Any	539	78.6	644	81.3	283	80.9	11,862	87.1
Hospital group								
NICU or CPAP	473	69.0	510	64.4	183	52.3	9404	69.0
Urban	112	16.3	117	14.8	56	16.0	2165	15.9
Regional	101	14.7	165	20.8	111	31.7	2054	15.1

*NICU* neonatal intensive care unit, *CPAP* continuous positive airway pressure; *SCN* special care nursery

^a^Percentages may not sum to 100% due to missing values

**Fig. 1:**
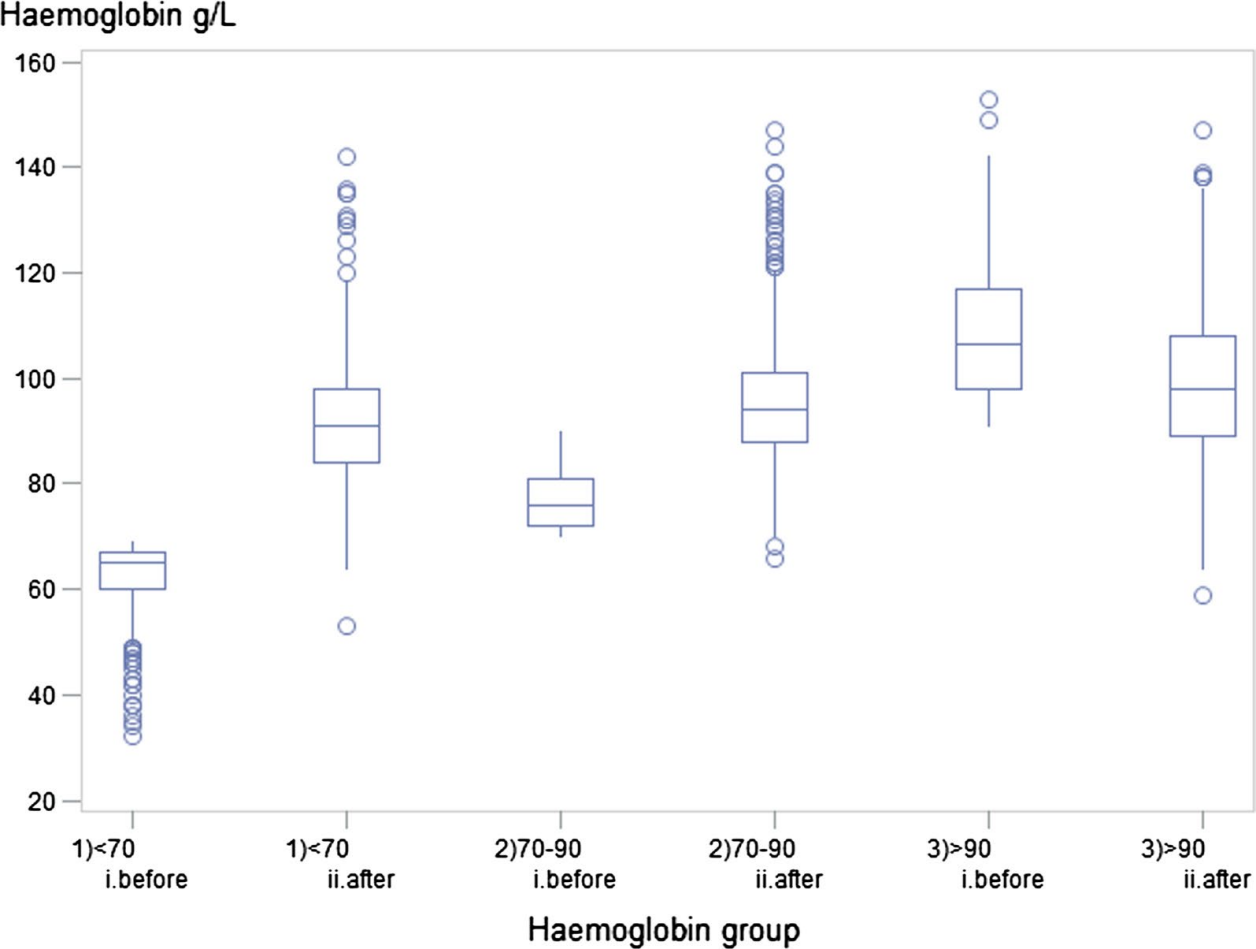
Haemoglobin concentration before and after transfusion by haemoglobin group before transfusion

Among the 1828 transfused mothers, 1714 (94%) had one or more haemoglobin measures after transfusion and before discharge. The median haemoglobin concentration before and after transfusion for each haemoglobin group was 65 and 91 g/L, 76 and 94 g/L and 107 and 98 g/L (Fig. 1). There was a significant increasing trend between haemoglobin group pre-transfusion and haemoglobin concentration post-transfusion (p < 0.0001). For those mothers with multiple haemoglobin measures after transfusion, the highest value was used to calculate these medians.

Eighty percent of transfused mothers were breastfeeding at discharge compared to 87% of non-transfused mothers. Among the transfused mothers, the percentage breastfeeding across the three pre-transfusion haemoglobin groups—< 70 g/L, 70–90 g/L, > 90 g/L—were 79%, 81% and 81% respectively (Table 1).

The adjusted relative risk for any breastfeeding for transfused women compared to non-transfused women was 0.90 (99% CI 0.86–0.95) for the haemoglobin < 70 g/L group, 0.94 (99% CI 0.90–0.98) for the haemoglobin 70–90 g/L group; and 0.94 (99% CI 0.88–1.00) for the haemoglobin > 90 g/L group (Table 2).

**Table 2:** Unadjusted and adjusted relative risks (uRR; aRR) for transfusion with any breastfeeding by haemoglobin concentration prior to transfusion

Variable	Value	n	Any breastfeeding	uRR (99% CI)	aRR^a^ (99% CI)
Transfusion	Haemoglobin < 70 g/L and transfusion	686	539 (79%)	0.90 (0.86–0.95)	0.90 (0.86–0.95)
	Haemoglobin 70–90 g/L and transfusion	792	644 (81%)	0.93 (0.89–0.98)	0.94 (0.90–0.98)
	Haemoglobin > 90 g/L and transfusion	350	283 (81%)	0.93 (0.87–0.99)	0.94 (0.88–1.00)
	No transfusion (reference)	13,623	11,862 (87%)	1.00	1.00

^a^Adjusted for all variables in Table 1 except for private obstetrician, placenta praevia/accreta, large and small for gestational age and resuscitation

### Discussion

We examined the association between red blood cell transfusion and breastfeeding in women with postpartum haemorrhage by differing postnatal haemoglobin concentrations. We found that red blood cell transfusion was associated with a reduced rate of breastfeeding and this was a consistent finding irrespective of haemoglobin concentration pre-transfusion. The median haemoglobin concentration post-transfusion was greater than 90 g/L for each haemoglobin-transfusion group, which suggests that severe persisting anaemia was not contributing to the reduced breastfeeding rates in the transfused groups.

The findings of this study align with a population-based analysis of breastfeeding rates at discharge in women with postpartum haemorrhage in NSW hospitals, which found that transfusion was associated with a lower rate of breastfeeding [9].

Among the strengths of our study is the study population selection. By defining the study cohort as women with postpartum haemorrhage, we were able to assess the impact of transfusion among a comparable group of women at risk of transfusion. Furthermore, by restricting the cohort to less severe postpartum haemorrhage and term infants, confounding of breastfeeding rates by haemorrhage severity and infant complications was reduced.

## Limitations

This study was limited by a lack of data on haemoglobin concentrations in women not transfused. Each haemoglobin-transfusion group was compared to the entire non-transfused group so breastfeeding at discharge may have been confounded by differences in haemorrhage severity or haemoglobin level. That is, the reason women were transfused may also be the reason they were less likely to breastfeed than women who weren’t transfused. However, even the highest haemoglobin-transfusion group, which is likely to have similar if not higher haemoglobin levels compared to the non-transfused group, had a reduced breastfeeding rate. Other study limitations include lack of available information on ongoing breastfeeding post-discharge and amount of blood lost or transfused. Given that some women are discharged from hospital within 48 h and before breastfeeding is established (and this is more likely to occur among the untransfused women), we may be underestimating the extent of the effect of transfusion on reduced breastfeeding. Finally, the decision for intrapartum transfusion may have been made based on point of care haemoglobin testing which can be less accurate than laboratory testing [20] and no information on duration of bleeding was available so it is difficult to draw conclusions about the appropriateness of transfusion from these data.

## Abbreviations

APDC: Admitted Patient Data Collection
aRR: adjusted relative risk
CEC: Clinical Excellence Commission
CHeReL: Centre for Health Record Linkage
CI: confidence interval
NSW: New South Wales
PDC: Perinatal Data Collection
uRR: unadjusted relative risk
